# Therapists Make the Switch to Telepsychology to Safely Continue Treating Their Patients During the COVID-19 Pandemic. Virtual Reality Telepsychology May Be Next

**DOI:** 10.3389/frvir.2020.576421

**Published:** 2021-01-15

**Authors:** Mariana Sampaio, Maria Vicenta Navarro Haro, Bruno De Sousa, Wilson Vieira Melo, Hunter G. Hoffman

**Affiliations:** 1 Department of Psychology, University of Coimbra, Coimbra, Portugal,; 2 Department of Social Work, Catholic University of Portugal, Lisbon, Portugal; 3 Instituto Aragonés de Investigaciones Sanitarias, Universidad de Zaragoza, Zaragoza, Spain; 4 Department of Psychology and Sociology, Faculty of Economics and Business, University of Zaragoza, Zaragoza, Spain,; 5 Faculty of Psychology and Educational Sciences, University of Coimbra, Coimbra, Portugal; 6 Independent Researcher, Porto Alegre, Brazil; 7 Department of Mechanical Engineering, Radiology and Psychology, University of Washington, Seattle, WA, United States; 8 Human Photonics Lab, Virtual Reality Research Center, University of Washington, Seattle, WA, United States

**Keywords:** COVID-19, virtual reality, mental health, telepsychology, burnout–professional, psychology, stress, anxiety

## Abstract

Before COVID-19, most therapists had concerns about telepsychology, and only treated patients in person. During the COVID-19 lockdown, patients still needed therapy, but in-person therapy sessions became unsafe. The current study measured how many therapists are using online therapy before vs. during COVID-19, how much training they have received, and their knowledge about legal restrictions on using telepsychology. A sample of 768 U.S.A. mental health professionals completed a 29-item online survey. Results show that before COVID-19, most therapists only saw their patients in person (e.g., at the therapists office), but during the COVID-19 pandemic, nearly all therapists used a wide range of telecommunication technologies to communicate with their quarantined patients, including texting, telephones, video conferences, and even virtual reality. According to within-subject related samples comparisons, 39% of survey respondents used telepsychology before COVID-19, vs. 98% during COVID-19 (χ^2^ = 450.02, *p*< 0.001). Therapists reported high treatment effectiveness using telepsychology (7.45 on 0–10 scale). However, overall, on a 0–10 scale, therapists reported a significant increase in feeling burned out during the COVID-19 pandemic, Mean = 3.93 (SD = 1.93) before vs. 6.22 (SD = 2.27) during the pandemic (*Z* = −18.57, *p* < 0.001). Although the APA ethics guidelines encourage therapists to use telepsychology with their patients during the crisis, gaps in respondents’ knowledge identify a need for increased specialized training and education. Although the current study showed that virtual reality is rarely used by the therapists surveyed, virtual reality is a promising new telepsychology technology. Billions of dollars are currently being invested in mass producing immersive virtual reality systems. In the future, as networked immersive Virtual Reality becomes more widely available, therapists and patients in physically different locations will be able to “meet” in a shared computer-generated world designed for therapy sessions, potentially including group sessions. Telepsychology and virtual reality have the potential to be increasingly valuable tools to help therapists mitigate the consequences of COVID-19. Research, development and training is recommended.

## INTRODUCTION

Healthcare is increasingly reliant on rapidly improving computer technology. For example, telepsychology has been successfully implemented in the Veterans Affairs (VA) system in the United States (Godleski et al., 2012; Caver et al., 2020) and remotely supported pain management services (Eaton et al., 2014; Eccleston et al., 2020). The expanding role of technology to help deliver psychological services and the continuous development of new technologies present unique opportunities, considerations, and challenges to practice (American Psychological Association, 2013). In a survey conducted by Glueckauf et al. (2018), nearly 40% of clinical psychologist in the USA reported using telepsychology, yet overall, ~60% never used telepsychology. They found that most clinical psychologists had concerns about telepsychology and only treated patients in person. Over 50% of the psychologists in the survey of Glueckauf et al. (2018) were concerned that they needed more skills on how to manage crisis situations during online therapy, and 79% of the therapists in their study had concerns about security, confidentiality, and Health Insurance Portability and Accountability Act of 1996 (HIPAA).

### Outbreak Forces Social Isolation

In March 2020, coronavirus disease 2019 (COVID-19) was declared a pandemic by the World Health Organization (World Health Organization, 2020a). Recent evidence suggests that the deadly virus is so contagious that one can catch COVID-19 by simply talking to an infected person (Ningthoujam, 2020), and 40% of infected people remain unaware that they are even sick, making it hard to know who to avoid. To reduce the number of people infected, in 2020, in the United States, social distancing laws have been implemented (Wellenius et al., 2020). Stay-at-home orders include restricted movement/minimal social interactions outside of your household, and nearly all businesses in the United States were forced by the government to close for months, with devastating economic and psychological impact (Atkeson, 2020).

### The Psychological Impact of COVID-19 and Social Isolation

Early studies indicate that the COVID-19 virus outbreak may be associated with increases in stress, fear, anxiety (Pierce et al., 2020), strong emotions, posttraumatic stress disorder (PTSD) symptoms (Tingbo, 2020), substance abuse (Rehm et al., 2020), and domestic violence (Mahase, 2020). Social distancing reduces social support from friends and relatives and negatively affects family dynamics (Christofferson et al., 2020; Prime et al., 2020), and many people feel lonely. A number of organizations are also predicting future psychological consequences of COVID-19 and are predicting an increased need for mental healthcare delivery, even after the COVID-19 virus is physically under control (Pfefferbaum and North, 2020; Singh et al., 2020).

People with pre-existing mental health conditions such as depression, anxiety, bipolar disorder, schizophrenia, or substance use disorders may have unusually strong reactions to crisis situations (CDC, 2020), especially with reduced social support. To avoid COVID-19 outbreaks, older adults living in nursing homes or assisted living condos are not allowed any visitors (no wives, husbands, children, friends, or family are allowed). Patients with COVID-19 who need to be hospitalized are also strictly isolated and only interact with healthcare givers who enter their hospital room wearing hazmat suits and protective respirator masks. COVID-19 patients die alone, are buried without funerals, and the family members are often unable to console each other in person during grieving of their lost loved one. Many healthy people are voluntarily self-isolating to help slow the spread of the virus. Children are spending less time with their friends, many schools are closed, and many children are home schooled, which can annoy children and parents alike. Millions of Americans are now unemployed, further stressing families. COVID-19 is a worldwide disaster. According to the CDC (2020), it is especially important for psychology patients to continue to receive clinical therapy during the crisis.

### Ethical Dilemmas

COVID-19 creates an ethical dilemma for therapists. The therapists have an ethical obligation to continue to help their patients during a crisis. The crisis may exacerbate the patient’s psychological symptoms (e.g., substance abuse, increased anxiety, etc.). However, therapists are also obligated to “do no harm,” so they cannot continue to meet their patients in person. One goal of the current survey was to see how therapists responded to this dilemma.

The American Psychological Association (APA) encourages therapists to use teletherapy to continue helping their patients during the crisis. Although many therapists are not fully trained to perform online therapy (Perry et al., 2020), the APA ethics code encourages psychologists to be flexible in emergency situations: “during times of crisis...Psychologists may provide services to those in need, even if they have not yet obtained the necessary training” (Chenneville and Schwartz-Mette, 2020, p. 3).

### Use of Telepsychology

In an earlier survey (Glueckauf et al., 2018), many therapists (43%) were already using telehealth, and most thought it was valuable and appeared to be open to the idea. For example, in the study by Glueckauf et al. (2018), 73% of clinical psychologists regarded videoconferencing as a useful tool for therapy, and 51% indicated that they would like to use telepsychology in the future. In the current study, we measured whether therapists switched from an in-person practice to using telepsychology so they could continue treating their patients during the COVID-19 social isolation.

### Telepsychology Training Needs

Previous research had suggested a need for training in telehealth in psychology graduate programs and continuing education after completing academic and vocational degrees (Callan et al., 2017). Glueckauf et al. (2018) found that around 90% of psychologists indicated that “mental health practitioners should undergo training about the clinical, legal, and/or ethical issues related to telehealth.” Most participants also reported the need of receiving training on technical issues surrounding delivery of telehealth services. Furthermore, in self assessments, around half of psychologists reported inadequate skills in managing emergency situations when using online counseling modalities, and around 40% also reported insufficient telehealth training or education. A recent study found that an important barrier to using telepsychology was therapists’ lack of self-efficacy due in part to insufficient opportunities for training (Perry et al., 2020). In the current study, we evaluate therapists’ ethical and legal/regulatory concerns related to delivery of telepsychology services and therapists’ views about the education and training needed to use telepsychology in clinical practice.

### Burnout

Therapists are considered frontline essential workers. During a worldwide major crisis like COVID-19, many or most of the therapist’s patients are having more psychological troubles and symptoms than usual, which makes the therapist’s job more stressful/demanding (e.g., helping one client after another deal with the increased stresses of social isolation during COVID-19). Furthermore, switching from in-person therapy to telepsychology may require just-in-time training for the therapists who were not using telehealth before COVID-19 (e.g., therapist may have to help their patients install the correct software application, reassure anxious patients about security issues, etc.). In the current survey, therapists rate how burned out they themselves felt before COVID-19 started and how burned out they are feeling now, during the COVID-19 pandemic. We predicted that therapists would report feeling significantly more burned out during the pandemic than they felt before COVID-19.

### Mindfulness

**FIGURE 1 |:**
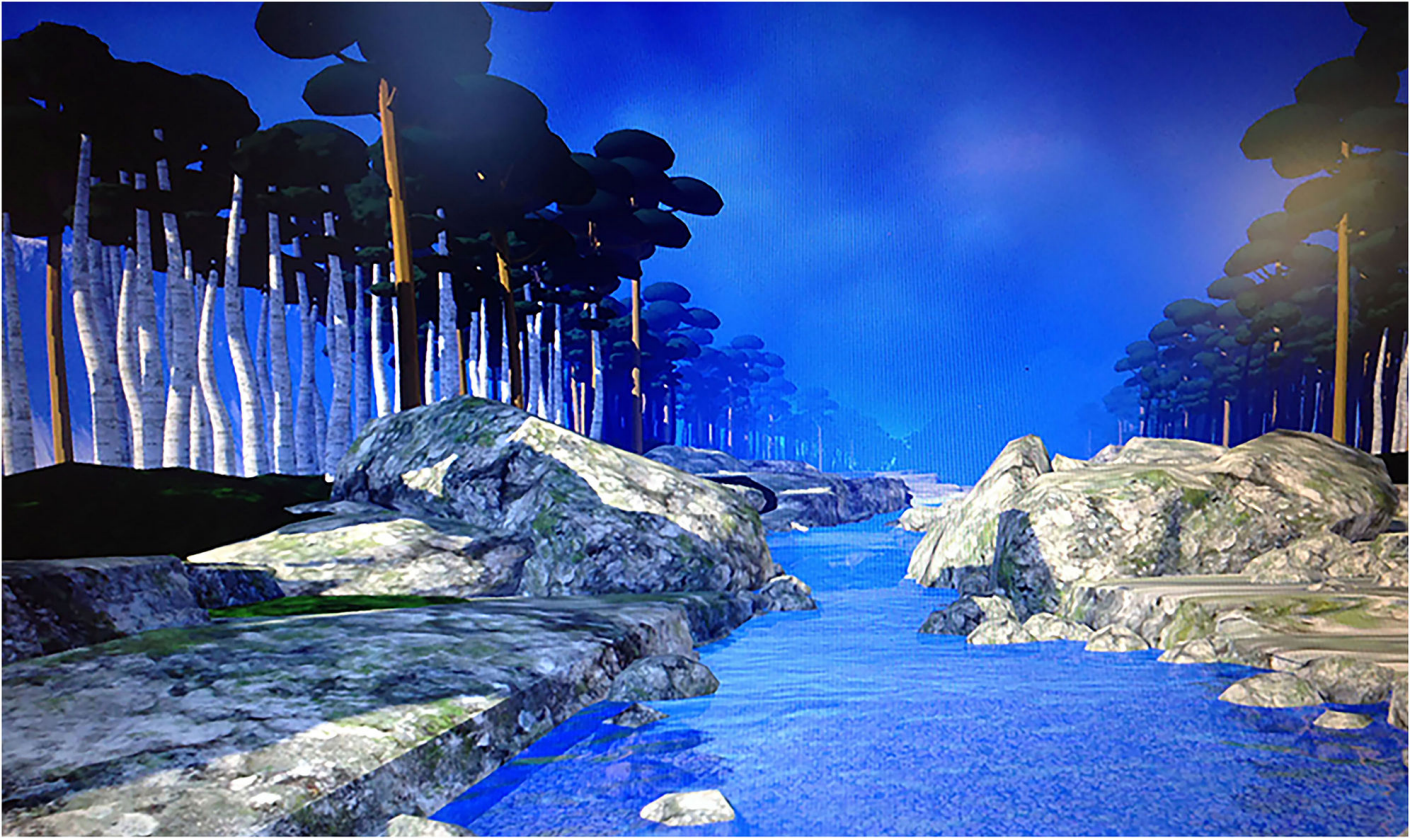
Mindfulness RiverWorld, image by http://www.bigenvironments.com/, copyright Hunter G. Hoffman, http://www.vrpain.com/.

Mindfulness practice can help reduce job burnout among healthcare professionals (Luken and Sammons, 2016). Mindfulness is one of a number of effective coping strategies that might help therapists deal with the increased stressors associated with the pandemic. Researchers have recently begun exploring virtual reality mindfulness skills training. Patients put on a virtual reality helmet and go into a computer generated world (e.g., RiverWorld, see Figure 1) where they receive training on how to practice mindfulness. Preliminary results suggest that Virtual Reality mindfulness training helps reduce stress, reduces negative emotions, and increases positive emotions, after the VR sessions (Navarro-Haro et al., 2016, 2017; Gomez et al., 2017; Flores et al., 2018). The current survey asks therapists about their personal use of mindfulness before vs. during the pandemic and their interest in trying VR mindfulness in the future.

### Networked Immersive Virtual Reality Therapy

Telepsychology began with landline telephone calls and has evolved to include cellphones and videoconferencing, as those newer technologies became available. Therapists using telepsychology are gaining access to increasingly sophisticated telecommunications technology. Improvements to the internet infrastructure and improved security of online communications have increased the opportunities for confidential communications with patients about sensitive topics due in part to successful efforts from the VA (https://telehealth.va.gov/). There is currently a multibillion dollar investment by major computer companies to get immersive virtual reality technology into peoples’ homes, e.g., immersive video gaming, immersive Facebook. Telehealth is projected to be the second largest use of networked immersive VR (Bailenson, 2018). The current survey asks therapists for the first time if therapists are already using immersive virtual reality to help treat their psychology therapy patients, and the current survey includes several exploratory questions to obtain an initial assessment of therapists reaction to the idea of using virtual reality during treatment. If the current trend is any indication, networked immersive VR therapy (and augmented reality therapy) may be used with increasing frequency in the future, (e.g., Dilgul et al., 2020) and it will be interesting to track whether therapists start using VR and, if so, the therapists’ ratings of the pros and cons of immersive therapy in future surveys.

The current study was conducted to measure how the COVID-19 pandemic was affecting therapists’ use of telepsychology. The specific objective of this study was to understand whether therapists adopted telepsychology during the COVID-19 pandemic and their attitudes toward using telepsychology. The survey also measured what types of telecommunication technologies therapists used (including virtual reality). We also aimed to investigate how much training therapists have had before vs. during the pandemic, therapists’ knowledge about legal restrictions on using telepsychology, need for more education, and ethical and regulator concerns about using telepsychology (security/confidentiality, HIPPA compliance, lack of training, and inability to handle emergency situations). Another goal was to understand how the pandemic affected mental health professionals by measuring whether therapist’s burnout had increased during the pandemic. The survey also measured therapists’ interest in using virtual reality mindfulness to help reduce their own stress, and we developed an open comments section, where therapists could make any comments they wanted, including comments about equity issues. We hypothesized that there would be a significant increase in use of telepsychology during social isolation, and we predicted that the results would also indicate areas for improvement in terms of therapists’ knowledge about rules and regulations of using telehealth. This is understandable considering that the virus hit the United States very fast and hard, with well over 330,000 American COVID-19 deaths between April and October 31, 2020, and the number of deaths projected to climb steeply in early 2021, so therapists had to switch very quickly and unexpectedly from in-person therapy to telehealth. Finally, we predicted that the therapists and patients in the United States would be willing and able to switch to telepsychology, to help their patients during this crisis, when therapy is especially important, but we predicted that some areas needing improvement would be identified. We predicted that therapists would report feeling significantly more burned out during the pandemic than they felt before COVID-19, and we expected an interest in using mindfulness to help therapists reduce their own stress.

## METHOD

### Participants

A sample of 768 English-speaking mental health professionals (therapists) completed a 29-item survey. Characteristics of the study sample (e.g., participants demographics, education) are described in Results.

The current investigation was approved by the University Institutional Review Board at the University of Washington, Seattle, and was preregistered at https://clinicaltrials.gov/ Identifier: NCT04360850. To be eligible to participate in the survey, participants had to be 18 years of age or older and currently active providing mental healthcare to patients (within the past year). Respondents were included in the study if they were (a) mental health professionals in the United States or its territories and (b) provided mental healthcare (e.g., clinical therapy or clinical counseling services, or clinical social workers). Potential participants were fully informed about the nature of the survey and anonymously clicked “continue to survey” if they were 18 years or older, had not completed the survey before, if they were actively working in their mental health field, and if they wanted to complete the survey and gave their informed consent to participate. To ensure anonymity, participants were not asked to disclose personal information and were assured that no personal data would be collected that could potentially identify them, such as email addresses.

### Procedure

This was an anonymous survey to be filled out online by mental health professionals (e.g., licensed clinical psychologists, social workers, mental health counselors, etc.). Prospective mental health professionals (i.e., therapists) were recruited by invitation and were provided with a link to a secure online survey located at www.limesurvey.org. These invitations were distributed to therapists in the United States via closed social media professional groups and electronic mailing lists from different boards, federations, and institutions associated with the mental health field, as well as peer to peer invitation.

The online survey took ~10–15 min to complete. The time frame of data collection was April 24th, 2020 to May 18th, 2020. The researchers subsequently downloaded the deidentified data from www.limesurvey.org to IBM SPSS Statistical Analysis System Version 25 (2019), for statistical analyses.

### Instruments

The survey titled “Use of telehealth technology by mental health care professionals in times of COVID-19 pandemic” developed for the current study was adapted from a 28-item survey by Glueckauf et al. (2018). The current survey focused on evaluating four key domains of using telehealth to deliver clinical services by professionals psychologists before the COVID-19 pandemic: providers’ use of telecommunication modalities; client population characteristics; professional, ethical, and legal/regulatory issues; and telehealth training and practice.

A number of questions of Glueckauf et al. were asked verbatim, and several other questions were modified/customized by our team to assess “before pandemic” vs. “during pandemic” changes in clients’ needs and therapists’ attitudes and behavior toward using telepsychology to conduct therapy sessions, and several new questions were added regarding the therapists self-ratings of burnout, use of mindfulness and virtual reality devices, the effectiveness of telehealth, and how much they are volunteering telehealth support.

In the current study, therapists filled out the survey at one time point and were retrospectively reporting on their therapy practices prior to the pandemic and also during the pandemic. For example, therapists were asked “Have you been working with telehealth/telepsychology before the novel coronavirus pandemic started (yes/no),” and the same therapist was asked “Have you been working with telehealth/telepsychology after the novel coronavirus pandemic started (yes/no).” It is not the case that data were collected at two different time points. The survey started with a demographic section (e.g., age, gender, ethnicity) followed by 29 True False/multiple-choice questions regarding current professional status (e.g., primary profession, work setting) and previous education (e.g., time since graduation). Professional therapists were asked about their (a) use of telepsychology before vs. during the COVID-19 pandemic; (b) changes in their recruitment and retention of patients before and during COVID-19; (c) types of telecommunication modalities (e.g., videoconferencing, email, virtual reality, etc.) used in clinical practice before vs. during the COVID pandemic, and types of populations and settings; (d) therapists’ burnout associated with delivering therapy before vs. during COVID-19; (e) therapists’ ethical and legal/regulatory concerns related to delivery of telepsychology services; (f) therapists’ views on education and training needed to use telepsychology in clinical practice; and (g) therapists interest in using immersive Virtual Reality therapy (VR mindfulness) in the future. In summary, our revised survey used in the current study is composed of 11 sociodemographic questions and 4 different domains regarding the use of telehealth technology by mental healthcare professionals: Health Professional’s Profile; Professional, Ethics, and Legal/Regulatory Issues; Telehealth Training; and Telehealth Practice (a complete list of survey questions is available). The survey ends with an open answer section allowing professionals to comment about any other relevant information regarding the survey topics.

### Data Analysis Strategy

Descriptive variables are presented using means (and standard deviations) for the continuous variables and frequencies (and percentages) for the categorical variables. Parametric and non-parametric statistics (*t*-tests, McNemar’s test, and Wilcoxon signed-rank test) were used for within- and between-subject paired and independent comparisons. IBM SPSS Statistical Analysis System Version 25 (2019) and R Project software version 4.0.3 (2020) were used to conduct data analysis.

## RESULTS

### Sample Characteristics

**FIGURE 2 |:**
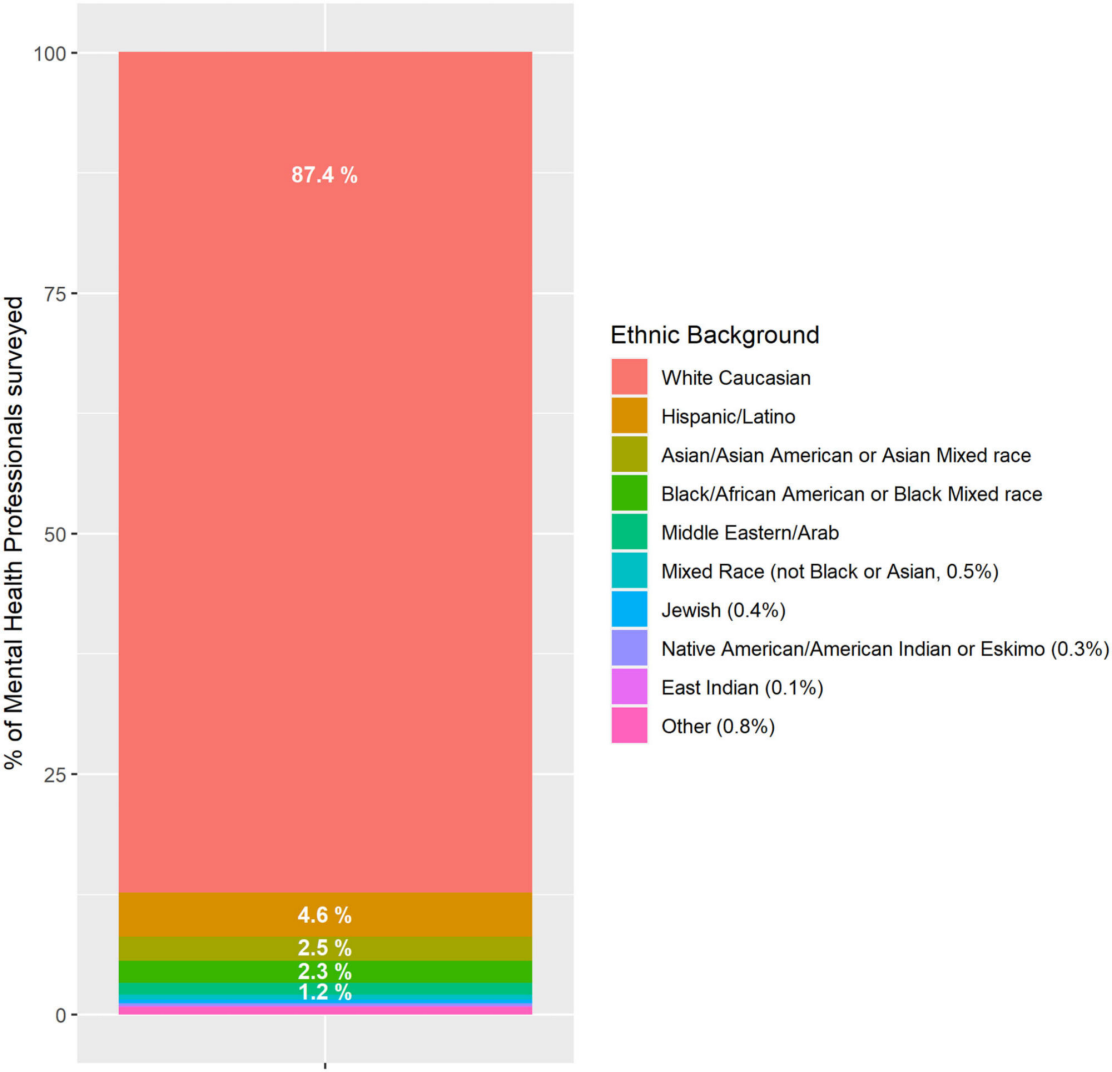
Ethnic background of therapist survey respondents.

The mean age of the respondents (professional therapists) was 43.74 years old (SD = 10.91). The gender was 92.2% female, 5.7% male, and 2.1% other. Ethnic background is described in Figure 2. The primary work settings were private practice = 80.59% (*n* = 619), public practice = 15.23% (*n* = 117), and other = 4% (*n* = 32). Private practice settings included independent practice, small group practice (2–9 practitioners), and large group practice (10 or more practitioners). Public practice settings consisted of health or behavioral health clinics, hospitals, military and university healthcare facilities, and the US Department of Veterans Affairs.

Most therapists had worked a number of years since obtaining their highest academic degree, mean = 12.02 years, SD = 8.78 years, (*n* = 758), range = 0–10 years, and had worked several years since licensure, mean = 9.62 years, SD = 7.97 years (*n* = 688), range = 0–43 years.

### Preference and Use of Telepsychology Before vs. During COVID-19

Non-parametric statistics for paired comparisons were used (McNemar test and Wilcoxon signed-rank tests). According to within-subject paired comparisons, less than half of the therapists (39% of survey respondents) used telepsychology before COVID-19, but nearly all therapists (98% of the 768 survey respondents) reported using telepsychology to treat their patients during the COVID-19 pandemic, a statistically significance increase (χ^2^ = 450.02, *p* < 0.001). The therapists surveyed reported a significant increase in specific training about telehealth, 38% before vs. 61% during COVID-19 (χ^2^ = 70.26, *p* < 0.001).

Therapists already using telehealth before the pandemic (early-adopter therapists) reported a pattern of better patient recruitment and retention during the pandemic compared to late adopters (i.e., therapist who only began using telehealth after COVID-19 social distancing measures began in the United States). Below are therapists’ responses to the following questions:

Since the novel coronavirus pandemic started, has the amount of need/requests for services from clients/ patients you already work with increased, decreased or stayed the same?”

**Table:** 

Requests from current patients	
Early-adopter therapists (*n* = 299)	late-adopter therapists (*n* = 467)
29.4% decrease	41% decrease in requests during COVID
36.5% increase	26% increase in requests
33% same	32.5% same

Since the novel coronavirus pandemic started, has the number of requests for services from new clients/patients: increased, decreased, or stayed the same?

As shown above, therapists who already used telepsychology before COVID-19 (i.e., early-adopter therapists) reported an overall increase in requests for therapy services from current clients, whereas late-adopter therapists reported an overall decrease in requests for therapy services from current clients (χ^2^ = 13.06, *p* = 0.001).

**Table:** 

Requests from new patients	
Early-adopter therapists (*n* = 299)	late-adopter therapists (*n* = 467)
45% decrease	53% decrease in requests during COVID
22.7% increase	17% increase in requests
31.1% same	29.8% same

As shown above, with regards to requests from new patients although non-significant, therapists who already used telepsychology before COVID-19 (early adopters) were better off than late adopters (χ^2^ = 4.899, *p* = 0.086, NS).

**FIGURE 3 |:**
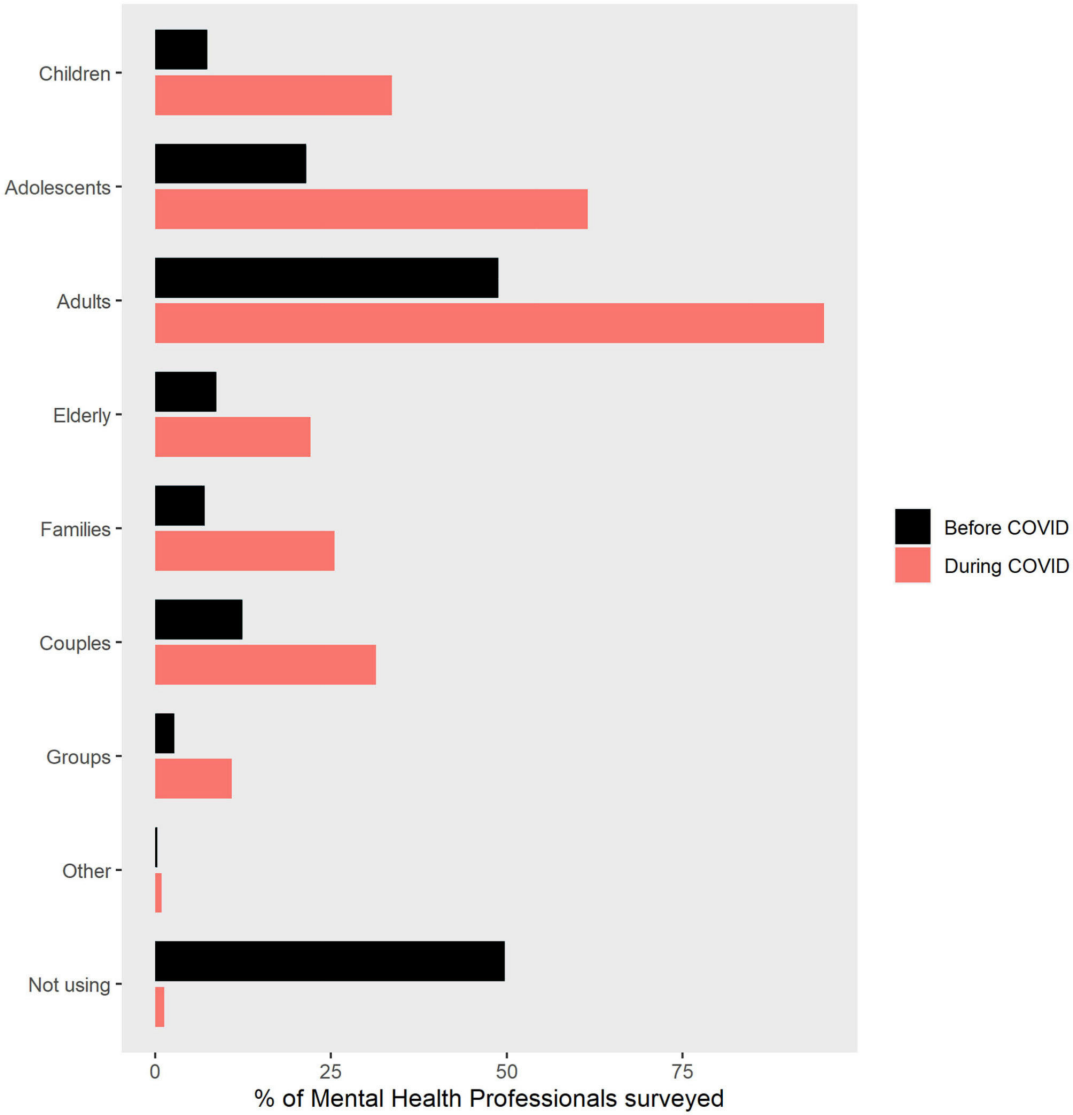
Percent increase in telepsychology during pandemic in all primary profession categories.

**FIGURE 4 |:**
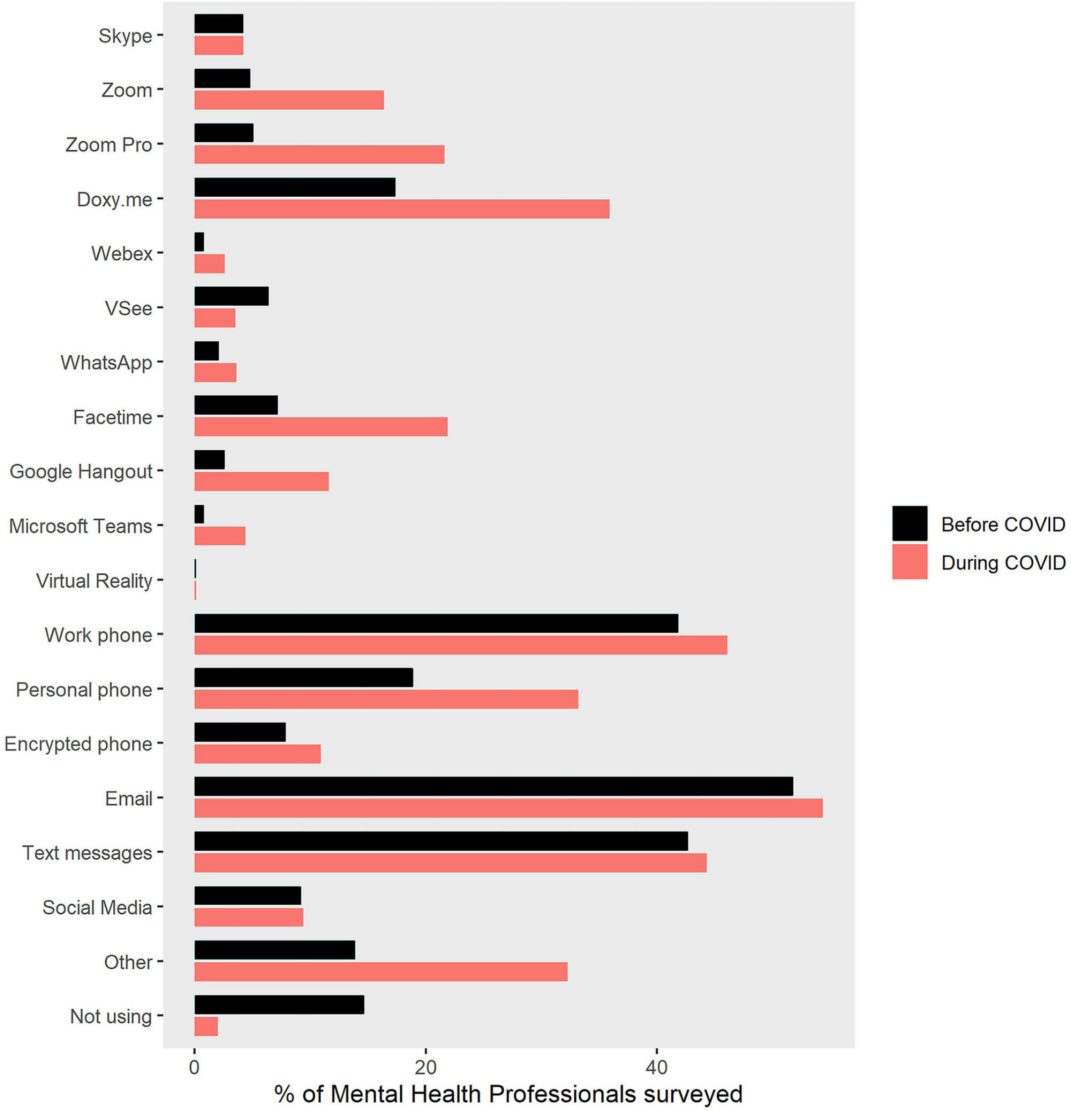
The most frequent modes of telecommunication used during teletherapy.

As seen in Figure 3, therapists reported large increases in the percentage of patients they treated via telepsychology during COVID-19 in all Primary Profession categories, including children, adolescents, adults, elderly, family therapy, couples, and groups. As shown in Figure 4, the most frequently used modes of telecommunication were work phone, email, and texting. Use of work phones, text messaging, and email with patients was high before the pandemic and stayed high during the pandemic. As shown in Figure 4, descriptive results of the current survey showed large increases in therapists’ use of several types of telepsychology technologies during the COVID-19 isolation. Use of Zoom free, Zoom pro, Doxy.me, Facetime, and personal phone use all increased during the pandemic. As shown in Figure 4, as measured in Spring 2020, Virtual Reality was rarely used by therapists to treat patients, and that was true both before and during the pandemic.

### Attitudes Toward Telepsychology Before vs. During COVID-19

**FIGURE 5 |:**
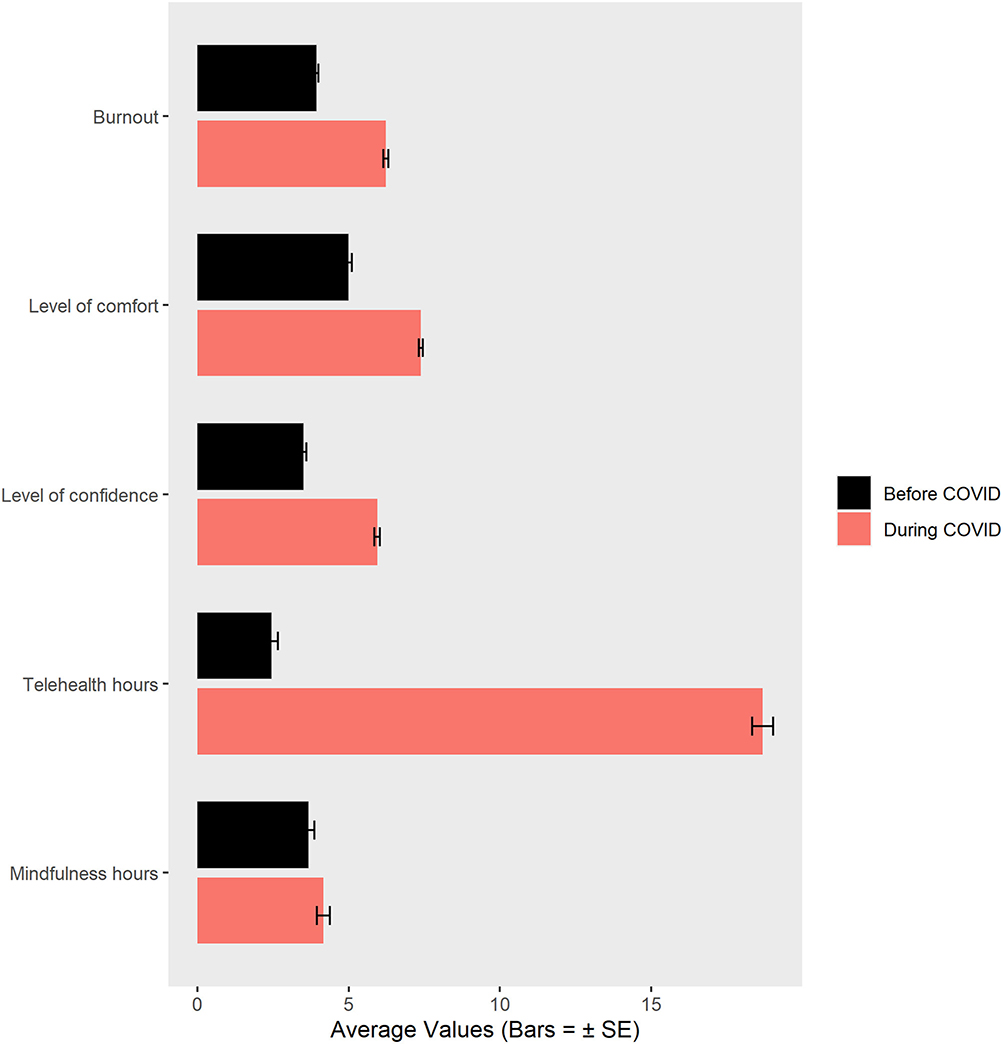
Wilcoxon signed rank paired comparisons of therapists’ comfort, confidence, burnout, and use of mindfulness, and percent of therapists using telepsychology before vs. during the pandemic.

The following four Wilcoxon signed-rank-related sample comparisons are shown in Figure 5. On a 0–10 scale, therapists reported becoming more comfortable using telepsychology during the pandemic, (*n* = 768), i.e., before COVID-19, mean = 4.99 (SD = 2.98) vs. during COVID-19, mean = 7.38 (SD = 1.95), Wilcoxon signed-rank test with *Z* = −18.14, *p* < 0.001. They also became more confident providing telepsychology without an initial in-person assessment (*n* = 768), before COVID-19, mean = 3.50 (SD = 2.90), vs. during COVID-19, mean = 5.94, (SD = 2.54), Wilcoxon signed-rank test with *Z* =−19.43, *p* < 0.001.

### Burnout

On our single item 0–10 graphic rating scale measure, measuring self-report perception of burnout according to Freudenberger’s definition, therapists reported a significant 37% increase in feeling burned out during the COVID-19 pandemic, mean = 3.93 (SD = 1.92) before vs. 6.22 (SD = 2.26) during the pandemic (*Z* =−18.57, *p* < 0.001). The subset of clinical social workers (*N* = 198) also reported a significant 37% increase in feeling burned out during the COVID-19 pandemic, mean = 3.98 (1.93) vs. 6.35 (2.21), Wilcoxon signed-rank test with *Z* =−9.68, *p* < 0.001.

### Mindfulness

Out of the 768 therapists in our sample, the subset of 468 therapists who reported personally using mindfulness for their own wellness reported a small increase in their own personal mindfulness practices during the pandemic: mean = 3.66 h/week (SD = 4.31 h) before vs. 4.17 h/week (SD = 4.68 h) during the pandemic, Wilcoxon signed-rank test with *Z* =−6.33, *p* < 0.001.

The 768 therapists surveyed reported high treatment effectiveness in using telepsychology. How effective do you think your telepsychology/telehealth services are? (0 = not effective at all, 10 = extremely effective), (*n* = 767), mean = 7.45 (SD = 1.61).

### Virtual Reality Mindfulness

Participants were asked the following exploratory question: “A growing number of people have been practicing Mindfulness/Meditation using virtual reality programs. If you have access to a virtual reality mindfulness program how many hours a week do you think you would practice mindfulness (on average)?” Out of the 768 therapists surveyed, 324 did not answer this question. Of the 474 who answered, 193 indicated they would not practice Virtual Reality mindfulness, and the remaining 281 therapists estimated that they would personally practice an average of 3.82 h of Virtual Reality mindfulness per week.

### Burnout, Mindfulness, and Virtual Reality

Comparing self-ratings of “burnout” in health professionals before the pandemic, we found no statistical differences (*t* = 1.803, *p* = 0.072) in the average levels of burnout between the individuals that practice mindfulness/meditation (3.84) and the ones that do not (4.09). During the pandemic, the differences between these two groups are also clearly not significance (*t* = 0.254, *p* = 0.800) with 6.25 being the average level of burnout for individuals that do not practice mindfulness/meditation, against 6.21 for the ones that do practice. It is worth noting that the increased average of 2.3 points in the level of burnout ratings by health professionals before and during pandemics was statistically significant (*t* = 26.078, *p* < 0.001).

When looking at the respondents (*n* = 474) who answered the question of being willing to try VR mindfulness stress reduction skills training, the average self-ratings of burnout are consistently higher for the individuals willing to try VR before the pandemics (4.05 vs. 3.76) and during the pandemic (6.38 vs. 5.97). Although these differences were not statistically significant (*p* = 0.111 and 0.051, respectively), the pattern of results suggest that therapists feeling more burned out might be more willing to try VR mindfulness.

### Professional, Ethical, and Legal/Regulator Issues

Therapists’ ethical and regulatory concerns about using telepsychology were also assessed (see Table 1). Most participants reported concerns regarding security/confidentiality or HIPPA Compliance and inability to handle emergency situations.

### Telepsychology Training

Respondents identified a need for increased specialized training and education in the future (see Table 1).

### Equity Issues

A number of equity issues arose in the comment section of our survey: some therapists raised concerns about equity, as many patients lose access to therapy services, as they lose their jobs and insurance benefits. Other inequities mentioned in the comment section include not having privacy for sessions (living in small houses with other family members, making it hard to speak freely to the therapist), not having proper access to technology, and therapists’ bias to physically avoid older patients more vulnerable to COVID-19.

## DISCUSSION

The current survey measured changes in therapists’ attitudes and behaviors toward using telepsychology to conduct therapy sessions during the COVID-19 pandemic. Specifically, in the current study, we measured whether therapists were using telepsychology so they could safely continue treating their patients during the COVID-19 isolation. Most therapists had a number of concerns about using telepsychology, including issues with security/confidentiality, HIPPA compliance, legality, lack of training, and inability to handle emergency situations online (e.g., suicidal patients). Before COVID-19, traditional therapists had the option of avoiding these issues by only treating patients in person. Results of the current survey showed that, before COVID-19, although there was a sizable proportion of “early-adopter” therapists already using telepsychology before the pandemic, 61% of the therapists in our survey only treated patients in person (not online) before the pandemic began. The current survey results show large changes in therapists’ behavior during the 2020 COVID-19 pandemic. Although only 39% of therapists used telepsychology before COVID-19, 98% of therapists surveyed were using telepsychology with their patients during the pandemic. A recent survey of licensed psychologists practicing in the United States found the same pattern: Use of telepsychology increased 12-fold to around 85% during the COVID-19 pandemic, with around 67% of psychologists conducting all of their clinical work with telepsychology during the COVID-19 pandemic at the time of the survey (Pierce et al., 2020).

How could such a huge shift occur so quickly? In addition to changes in therapists attitudes and behavior towards telepsychology, a number of legal and financial barriers to using telepsychology (and telehealth) were deliberately removed in early 2020. As described by Pierce et al., 2020, several influential medical centers took major early forward thinking steps in emergency response to the emerging COVID-19 crisis (e.g., already fully published changes in March 2020). https://www.hopkinsmedicine.org/coronavirus/articles/telemedicine.html; https://www.facs.org/covid-19/clinical-guidance/elective-case; https://www.va.gov/opa/docs/VHA_COVID_19_03232020_vF_1.pdf

In response to COVID-19, in early 2020, in addition to other changes, Veteran’s hospitals and Medicare temporarily enabled therapists to get reimbursed for telemedicine visits, at the same billing rate as in person visits (Pierce et al., 2020). In addition, according to Pierce et al., (2020, p. 2), “(HIPAA) regulations were eased by the United States Department of Health to allow use of apps like Skype, FaceTime, and Zoom.” Finally, several states loosened restrictions to allow therapists from other states to treat patients in their states during the COVID-19 pandemic (Pierce et al., 2020).

### Ethical Dilemmas

Treating patients in person during COVID-19 raises ethical dilemmas. According to the American Psychological Association (APA) Ethics code (Chenneville and Schwartz-Mette, 2020), during the COVID-19 global health crisis and its aftermath, therapists have an ethical responsibility to continue helping their clients, colleagues, and trainees, and to do no harm (Principle A), e.g., avoiding in-person therapy sessions during a virus pandemic, and therapists should also make reasonable adjustments in their practices to meet the clients’ mental healthcare needs (Principle B). In other words, APA recommends that mental health professionals should start using (or continue to use) telepsychology to treat their patients during COVID-19.

According to Chenneville and Schwartz-Mette (2020) (p. 8), “psychologists are permitted (and, some might argue, encouraged) to make reasonable extensions of their practice, even including working beyond the identified limits of their competence, to provide services in emergencies if they are able.”

Consistent with the APA Ethics code, during the COVID-19 pandemic, despite therapists’ concerns about being underprepared to use telepsychology, nearly all of the therapists who participated in the current survey used online therapy so they could continue treating their socially isolated “stay-at-home” patients. This result is promising given that over half of the therapists only began using telepsychology during the COVID-19 pandemic. Most therapists reported confidence using telepsychology and rated their therapeutic effectiveness using online therapy as 7.5 out of 10 (where 10 = extremely effective).

### Burnout

Although telepsychology has allowed therapists to continue helping their patients, COVID-19 has also been stressful for therapists. Freudenberger (1974) defined burnout to be a “state of mental and physical exhaustion caused by one’s professional life.” On our new single item rating of burnout before vs. during COVID-19, results of the current survey showed a statistically significant 37% increase in therapists’ self-ratings of “burnout” during the pandemic. Several therapists mentioned that their patients were having higher than usual difficulties during the pandemic. This may have contributed to the therapist’s increased burnout ratings, and therapists also mentioned that, in addition to having an unusually high percentage of their patients in crisis, the therapists were also dealing with their own isolation-related difficulties/issues, fears, and anxieties elicited by the pandemic [see also Prime et al. (2020)].

### Mindfulness

According to Chenneville and Schwartz-Mette (2020), it is important for mental healthcare providers to acknowledge their own sources of stress during the pandemic, and to take steps to minimize the negative impact of these stressors [see American Psychological Association (2013) recommendations]. “Psychologists should remember their ethical duty to care for themselves and their colleagues, so that they are able to care for those in need” (Chenneville and Schwartz-Mette, 2020, p. 3). In the future, therapists could likely benefit from learning more psychological techniques (e.g., mindfulness) to reduce their own stress and burnout (Luken and Sammons, 2016) during stressful worldwide crises. In the current survey, more than half of the therapists reported practicing mindfulness to help cope with their own stress, and there was a significant increase in the number of hours/week therapists practiced mindfulness during the pandemic. For patients and therapists alike, it is important to have good sleep hygiene, have regular exercise, eat healthy food, and avoid the temptation to use drugs and alcohol to help cope with the stress (Chenneville and Schwartz-Mette, 2020, https://www.cdc.gov/violenceprevention/suicide/copingwith-stresstips.html).

### Telepsychology Training and Education

In the current survey, most therapists (85%) indicated that it would be valuable to increase therapists’ access to training on how to use telepsychology with their patients, including training about laws and regulations of telepsychology, the need to use secured online communications, and training on how to deal with emergencies such as patients becoming suicidal. Courses on these topics are available online, and there is considerable helpful information on these topics online [e.g., APA.org, Chenneville and Schwartz-Mette (2020)]. Most of our survey respondents did not receive any training on telepsychology during their academic years. Furthermore, 85% of respondents recommended increasing future efforts to include telepsychology training into students’ college and upper division coursework. Continuing medical education courses are also recommended [see also Eaton et al. (2014)].

### Equity Issues

In the comment section of our survey, some participants reported challenges to working with children using telepsychology, and one therapist reported feeling that they were placed in a position of a “babysitter” for parents struggling for time, rather than providing therapeutic work. This is a good example of the need for more access to just-in-time online training on best practices for providing telepsychology to special populations such as children, as therapists step out of their comfort zones to help out a wider range of patients during the crisis. In other words, therapists need access to easy to understand, well-targeted educational information so they can quickly ramp up their knowledge in areas likely to arise in therapists who are just beginning to start using telepsychology. For example, therapists can go online and watch training videos from trusted sources such as the American Psychological Association, to help prepare the therapists to be as effective as possible in delivering telepsychology (e.g., tips for treating adolescents, for therapists who usually only treat adults). In addition, therapists raised concerns about equity, as many clients may lose access to therapy services, as they lose their jobs and insurance benefits. Over 40 million Americans have applied for unemployment so far, during the first half of 2020 (https://www.nytimes.com/2020/05/28/business/unemployment-stock-market-coronavirus.html). Other inequities include not having privacy for sessions (living in small houses with other family members), not having proper access to technology, and therapists’ bias to physically avoid older patients more vulnerable to COVID-19. In order to better assess increased inequity due to lack of access to mental health services, further research and advocacy in this area is recommended.

### Limitations

The current study has several limitations to consider when interpreting these results. Most survey participants were female (92.2%) and Caucasian (87.4%). Although similar demographic characteristics have been found in other studies with mental health professionals samples from the United States (e.g., Navarro-Haro et al., 2019a), the current study surveyed a voluntary sample of self-selected therapists, which may or may not accurately reflect the demographics, attitudes, and behaviors of therapists in the United States. Considering that COVID 19 is a pandemic (i.e., worldwide), future studies should collect data worldwide, not only data coming from the United States. Future studies should also include traditional measures of burnout whose validity and reliability have already been established. The Maslach Burnout Inventory (MBI) is a well-validated, widely used self-survey measure (Dolan et al., 2015). Encouragingly, Dolan et al. (2015) developed a single-item measure of burnout and found it to be a viable non-proprietary alternative that was highly correlated with the MBI. However, the single-item burnout measure used in the current study has not yet been validated, so the burnout results in the current survey should be interpreted with caution. Another limitation is that the current survey asked therapists about whether they are using mindfulness as a coping strategy to help reduce their own stress and burnout. The current research team is especially interested in mindfulness, but future surveys should also include questions about a number of other effective coping strategies, to see if any of the coping strategies are especially helpful for reducing stress and burnout of therapists (e.g., getting plenty of sleep, regular exercise, healthy food, avoiding the temptation of using alcohol, or drugs as a coping mechanism).

Another limitation is that therapists filled out the survey at one time point and were retrospectively reporting on their therapy practices prior to the pandemic and comparing that to their current therapy practice during the pandemic (the survey was taken in Spring 2020). For example, therapists were asked, “Have you been working with telehealth/telepsychology before the novel coronavirus pandemic started (yes/no),” and the same therapist was asked, “Have you been working with telehealth/telepsychology after the novel coronavirus pandemic started (yes/no).” It is not the case that data were collected at two different time points.

Finally, based on the results of our exploratory factor analysis (see Appendix), for future surveys, we recommend that all items and response choices of surveys should use interval or ratio scales, or at the very least, ordinal, avoiding nominal categories and particularly multiple choices questions (McDonald, 1999). Furthermore, although survey questionnaires are a valuable exploratory descriptive method, the use of more refined interviews and self-report measures as well as randomized controlled clinical and laboratory follow-up studies is needed to better understand the research topics addressed in the current study and to test new treatments for the psychological consequences of the COVID-19 pandemic. It would be interesting to investigate whether therapists’ burnout ratings are associated with mindfulness (or other self-care strategies) or to investigate which factors were associated with burnout.

### Future Psychological Consequences of COVID-19

Frontline healthcare professionals dealing with the sick and dying people with COVID-19 are reporting high stress, anxiety, depression, distress, and insomnia (Lai et al., 2020), and there are concerns about potentially large increases in depression and suicides in the United States, in both healthcare professionals and in the general population, in the aftermath of the pandemic and associated major worldwide financial downturn and increased societal instability. According to a recent literature review, “large scale disasters, whether traumatic (e.g., the World Trade Center attacks or mass shootings), natural (e.g., hurricanes), or environmental (e.g., Deepwater Horizon oil spill), are almost always accompanied by increases in depression, posttraumatic stress disorder (PTSD), substance use disorder, a broad range of other mental and behavioral disorders, domestic violence, and child abuse” (Galea et al., 2020, p. E1). WHO and other healthcare organizations are predicting that there will be a large worldwide increase in mental health issues for people who survive the pandemic, and they recommend recruiting and training more mental healthcare professionals to help mitigate the long-term consequences of the COVID-19 pandemic (World Health Organization, 2020b). Computer-assisted therapy tools could help.

### Future Directions: Immersive VR Telepsychology

The current results show a large increase in therapists’ use of computer-augmented clinical therapy (e.g., telepsychology) in the United States. On the related topic of computer-augmented therapy, there is a growing literature of studies exploring the use of immersive virtual reality to enhance the effectiveness of standard psychotherapy treatments such as cognitive behavioral therapy, dialectical behavioral therapy, mindfulness skills training (e.g., Carlin et al., 1997; Difede and Hoffman, 2002; García-Palacios et al., 2002; Hoffman, 2004; Difede et al., 2014; Navarro-Haro et al., 2016, 2017, 2019b; Reger et al., 2016; Gomez et al., 2017; Rizzo and Shilling, 2017; Flores et al., 2018; Boeldt et al., 2019; Katz et al., 2020), and virtual reality analgesia (Hoffman, 1998; Hoffman et al., 2000, 2019; Keefe et al., 2012). Virtual Reality therapy is proving valuable for assessing, preventing, and treating psychological problems including stress-related psychopathological problems, depression, anxiety disorders, e.g., phobias, social anxiety disorders (Anderson et al., 2005), generalized anxiety disorder, (Navarro-Haro et al., 2019a,b), and posttraumatic stress disorder (Rizzo and Bouchard, 2019). VR can help therapists and patients learn new psychological coping skills to help them be more resilient during and after the extreme stress levels elicited by the COVID-19 pandemic. Despite encouraging preliminary clinical research results on Virtual Reality therapy in the scientific literature and growing use of VR exposure therapy for PTSD (e.g., Rizzo and Shilling, 2017), the current survey suggests that Virtual Reality technology has not yet been widely adopted into mainstream clinical practice in the United States. In the current survey, only 1 out of 768 respondents indicated that they had used virtual reality technology to help treat their patients, and a number of respondents expressed interest in trying immersive virtual reality mindfulness to reduce their own stress. Until recently, VR technology has been too expensive and too difficult to use for most clinical therapists and has been primarily single player/not networked. However, as the result of recent multibillion dollar investments into immersive virtual reality technology (primarily for entertainment) by large computer companies, Virtual Reality systems are quickly becoming much less expensive and much easier to use (Bailenson, 2018). The essence of immersive virtual reality is the computer user’s illusion of “being there” in the virtual world, as if the computer-generated environment is a place they are visiting (Slater et al., 1994). In the near future, with networked immersive virtual reality, therapists and patients in different physical locations will be able to “meet” and “be there together” in a shared computer-generated world designed for therapy sessions, including one-on-one sessions and group therapy sessions (e.g., Dilgul et al., 2020). Future studies may explore the use of new multimodal clinical telepsychology therapy treatment approaches that include networked shared immersive virtual reality technology.

## CONCLUSION

In conclusion, this online survey helps understand changes in telepsychology adoption and changes in therapist’s attitudes and behavior toward using telepsychology to conduct therapy sessions during the COVID-19 pandemic. The current results indicate that there has been a significant increase in the use of telepsychology to conduct psychotherapy during the COVID-19 pandemic by mental health professionals in the United States, and now that they are using telepsychology regularly, most therapists surveyed now feel more comfortable using telepsychology with their patients even without an initial “in-person” assessment. However, a number of therapists still had ethical, training, and personal concerns regarding the use of telepsychology, and gaps in therapists’ knowledge on these topics were evident, indicating a strong need for increased telepsychology training for therapists in the future. The current study also identified a major increase in therapists’ burnout during COVID-19, suggesting that therapists could benefit from additional training on their own stress-coping skills during a crisis [e.g., practicing mindfulness while in a computer generated virtual world, Navarro-Haro et al. (2016), see Figure 1], and a number of other coping strategies could also be valuable. Finally, COVID-19 will likely increase the number of therapists needed in the USA and worldwide (World Health Organization, 2020b) to help mitigate the long-term psychological and economic consequences of the pandemic (Pfefferbaum and North, 2020; Singh et al., 2020). If there is a growing gap between the number of patients and the availability of highly trained therapists, and economic considerations during a damaged economy, computer-augmented therapy may be a cost-reducing way to help increase dissemination of effective psychological stress reduction treatments. Research and development of “COVID-19 therapy” is needed.

## Figures and Tables

**Table 1|:** Therapists’ ethical and regulatory concerns about using telepsychology and telepsychology training needs.

Survey on professional, ethical, and legal regulatory issues on telehealth training and practice (*n* = 768)				
	Yes % (*n*)	Unsure % (*n*)	No % (*n*)	Other % (*n*)
Does your malpractice carrier cover you for delivering telehealth services?	66.7 (512)	29.3 (225)	0.3 (2)	3.8 (29)^a^
Do you think it is ethical for licensed mental health professionals to deliver services online or via other telehealth technologies?	95.7 (735)	3.4 (26)	0.8 (6)	
Do you believe it is legal to practice over state lines or national borders using telecommunications technology if you call yourself a “coach”?	21.5 (165)	35.0 (269)	43.5 (334)	
Do you think it is legal for licensed professionals to provide services online to someone who is located in a state or country in which you are NOT licensed?	11.7 (90)	13.4 (103)	74.9 (575)	
Do you think mental health practitioners should undergo any training about: [The technical issues of telehealth]	87.1 (669)	6.6 (51)	6.3 (48)	
Do you think mental health practitioners should undergo any training about: [Clinical, legal, and/or ethical issues in telehealth]	97.5 (749)	1.4 (11)	1.0 (8)	
Do you think the average mental health professional can effectively screen for “at risk” clients (dangerous to self or others) using telehealth technology?	60.0 (461)	30.2 (232)	9.8 (75)	
Are you aware of any state or federal law/s or regulation/s that govern the delivery of counseling services provided online or via other telehealth technologies?	74.6 (573)	–	4.9 (38)	20.4 (157)^b^
Are you certified to work with telepsychology/telehealth?(Please remember that this survey is anonymous and that most places do not require certification in telepsychology/telehealth in order to practice it)	17.3 (133)	–	82.7 (635)	
Did your Undergraduate or Graduate University program offer telepsychology/telehealth specific classes or content?	4.3 (32)	–	95.7 (717)	
Do you think that it would be helpful for professionals to have training on telepsychology/telehealth as a part of their professional Undergraduate university program or Graduate University program?	90.4 (694)	–	9.5 (73)	
**What are your concerns about telehealth? (Select all that apply)**				
Security/confidentiality or HIPAA compliance	50.9 (391)	–	49.1 (377)	
Equipment costs	13.5 (104)	–	86.5 (664)	
Licensure issues	24.6 (189)	–	75.4 (579)	
Lack of personal training or education in this area	29.6 (227)	–	70.4 (541)	
Lack of direction from my professional association	13.3 (102)	–	86.7 (666)	
Lack of available education or training programs in this area	11.7 (90)	–	88.3 (678)	
Lack of supporting research	16.9 (130)	–	83.1 (638)	
Inability to handle emergency situations	48.7 (374)	–	51.3 (394)	
Tried online therapy practice and decided it was not for me	3.8 (29)	–	96.2 (739)	
Don’t know how to get started with online practice	1.4 (11)	–	98.6 (757)	
Don’t understand issues involved with online practice	2.3 (18)	–	97.7 (750)	
Don’t have any concerns?	8.7 (67)	–	91.3 (701)	
Have you been volunteering your services online to help people through the novel-coronavirus pandemic?	21.7 (167)	–	78.3 (601)	

a Do not have malpractice insurance.

b Some of them.

## Data Availability

The raw data supporting the conclusions of this article will be made available by the authors, without undue reservation. Requests to access the datasets should be directed to hunthoff9@gmail.com.

## APPENDIX

### Exploratory Factor Analysis of the Measures

The results of this exploratory factor analysis identified a number of limitations in the measures used in the current survey. Since this was an adaptation of the survey of Glueckauf et al. (2018), many of the items of both surveys are binary, multiple choice questions, and mostly with nominal type of answers. This poses a challenge when applying exploratory factor analysis (EFA) since it is recommended when creating a survey that the items and response choices should be considered interval or ratio or, at the very least, ordinal, avoiding nominal categories, and particularly multiple choices questions (McDonald, 1999). To overcome this challenge, our multiple choice questions (Q2, Q10, Q19, and Q26) that were very clearly identified with the domains under study were not included in our EFA-related calculations, and we used the package polycor from R to determine the correct matrix of association of the remaining questions through the function hector. This function allows us to calculate Pearson correlations (for numeric data), polyserial correlations (for numeric and ordinal data), and polychoric correlations (for ordered or non-ordered factors—the majority of our items). The EFA was then applied to the correlation matrix generated from this function, applying a VARIMAX rotation and using the function factanal. Several models from two to six factors were produced, explaining a percentage of variance ranging from 26.5 to 46.9%. After analyzing the results, we selected the model with four factors since not only it concurs with the theoretical concept proposed for the survey, but also this model would explain 40.3% of the variance. However, the goodness of fit of the model was clearly rejected ($\chi_{321}^{2}=21,018.59$, *p* < 0.001). After removing iteratively items that load in more than one factor (cross-loadings) and with low communalities, we obtain the final model: The percentage of variance explained in this model is 62.3%, and although an improvement from the first model, we still reject the goodness of fit of the ($\chi_{51}^{2}=13,084.38$, *p* < 0.0001)

**Table:**

	Factor 1	Factor 2	Factor 3	Factor 4
Q16 (before)	0.823			
Q16 (after)	0.649			
Q17 (before)	0.809			
Q17 (after)	0.642			
er Q1 (aft)		0.974		
Q20 (after)		0.712		
Q24		−0.593		
Q27		0.653		
Q9 (technical)			0.821	
Q9 (clinical)			0.992	
Q14			−0.602	
Q21 (before)				0.982
Q22 (after)				0.978
Q18	0.477			
Q20 (before)	0.398			

It is interesting to note that clearly factor 1 can be labeled as “Telehealth Practice;” factor 2 relates to workload, volunteering, and legal worries, call it “Work Issues;” factor 3 is regarding “Telehealth Training;” and finally factor 4 deals with the practice of “Mindfulness” before and after COVID-19. The results also suggest the need of some revisions of the items for future surveys, probably suggesting the use of response choices in interval or ratio measurements for future surveys.
